# Does the Personality of Consumers Influence the Assessment of the Experience of Interaction with Social Robots?

**DOI:** 10.1007/s12369-022-00935-5

**Published:** 2022-10-09

**Authors:** Santiago Forgas-Coll, Ruben Huertas-Garcia, Antonio Andriella, Guillem Alenyà

**Affiliations:** 1 Business Department, University of Barcelona, Avda. Diagonal, 690, 08034 Barcelona, Spain; 2 Institut de Robòtica i Informàtica Industrial CSIC-UPC, C/ Llorens i Artigas 4-6, 08028 Barcelona, Spain

**Keywords:** Social robot, Consumer personality, Front-office services, Social intelligence

## Abstract

In recent years, in response to the effects of Covid-19, there has been an increase in the use of social robots in service organisations, as well as in the number of interactions between consumers and robots. However, it is not clear how consumers are valuing these experiences or what the main drivers that shape them are. Furthermore, it is an open research question whether these experiences undergone by consumers can be affected by their own personality. This study attempts to shed some light on these questions and, to do so, an experiment is proposed in which a sample of 378 participants evaluate a simulated front-office service experience delivered by a social robot. The authors investigate the underlying process that explains the experience and find that cognitive-functional factors, emphasising efficiency, have practically the same relevance as emotional factors, emphasising stimulation. In addition, this research identifies the personality traits of the participants and explores their moderating role in the evaluation of the experience. The results reveal that each personality trait, estimated between high and low poles, generates different responses in the evaluation of the experience.

## Introduction

In recent years, there has been an increase in the presence of social robots in service organisations as equipment that helps control labour costs and improves both service enjoyment and the customer experience [1, 2]. But the outbreak of the Covid-19 pandemic, with the requirements of social distance and physical isolation, have accelerated its development and implementation, with 66 types of robots being used in 35 different countries, led by China (28%), USA (12.3%) and Thailand (10.2%) [3]. In some cases, social robots have replaced human employees in tasks that required high between-people contact (such as robot receptionists) while in others, they have offloaded them from less essential tasks (such as the robot that disinfects rooms in a hospital) [4]. One of the sectors hardest hit by Covid-19 has been the health and care sector, where, despite the use of special protective equipment, it is estimated that between 3 and 20% of all cases diagnosed were registered among their workers (by country: USA 3%; China 3.8%; Holland 6%; Italy 10%; Spain 20%) [5].

Although the industrialisation of services, with the implementation of new technologies, self-service machines or online systems, has changed the customer experience model, the consequences have not always been entirely positive [6, 7]. For example, the implementation of automated teller machines (ATM) is requiring a longer period of time than expected, since some users show difficulties in following the sequence of commands required for banking operations and they often get stuck and do not manage to complete them [8]. The technological innovation represented by social robots in services, thanks to their endowment of social skills, is not expected to require as long a deployment period as previous technologies [9]. To meet this challenge, several lines of research are being worked on, ranging from the design and development of mechanoid robots (e.g. drug dispensing robots in pharmacies) and chatbots (e.g. conversation devices) to humanoid and/or android robots to serve customers in service organisations [10].

A social robot is any robotic device with the ability to interact in social manner with humans. For example, a study comparing the effectiveness of a robot’s physical presence versus its on-screen view in providing personalized assistance to consumers, concludes that physical embodiment enhances learning and, therefore, its absence could be a handicap for its future development [9]. On the other hand, evidence has also been collected showing that the hasty use of androids (human-like forms) has given rise to the so-called "Uncanny valley" hypothesis, a situation of disappointment derived from the mismatch between the human form of the robot and the clumsy human abilities it manifests [11]. Although much progress has been made in the design and development of Artificial Intelligence (AI) systems to provide robots with social skills, there is still a long way to go. It seems that, in the short and medium term, robotic solutions will be focused on the design and development of humanoid robots (with simplified human features) with the ability to perform simple or complex cognitive-analytical tasks along with simple socio-emotional ones and, to cover more complex socio-emotional services, they will have to act jointly with human employees [10]. Some examples of commercialised humanoid robots, such as ARI and Pepper, are endowed with the ability to interact socially with humans using verbal and non-verbal social cues. This ability, albeit pre-programmed in the robot, makes the interlocutors perceive them as human [7], attribute personality to them and believe that they are similar to them [12, 13], and even to express attraction to them [14, 15].

To date, the literature has studied the effects of HRIs on the technological acceptance of social robots that display gender identity and personality traits in both in the social robotics [16–18] and the service domains [15, 19, 20]. However, it is not clear how consumers value these experiences or what the main drivers that shape them are. Furthermore, it is also unclear whether these consumer experiences may be affected by consumers’ own personalities. Therefore, two research questions are proposed.

- RQ1: What are the main factors that explain the valuation of the experience of receiving a service by a social robot?
- RQ2: How do consumers' personality traits affect the valuation of that experience?

To shed some light on these questions, this study proposes an experiment that simulates the provision of a front-office service by a social robot. In addition, the results of estimating that experience and the main drivers that explain it in a sample of users are presented, and analyses were performed to explore whether the experience they underwent is valued differently by the different consumer personality profiles (Extraversion, Agreeableness, Conscientiousness, Neuroticism, Openness). That is, if users with high personality traits, have the same evaluation of the experience as those with low traits in each of the five big personality traits [21], and how it affects the precedents that explain it.

When dealing with these issues, three contributions are made to the literature. First, although previous studies have assessed the HRI experience (a review is proposed by Shourmasti et al. [22]), our research is among the first to make the assessment using a structural model after experiencing interactions with a real social robot in a front-office service environment. Although there are some precedents in hospitality, such as Tung and Au [23], who studied the reviews published by customers on TripAdvisor, Booking.com, etc., where they rated their experience of staying in hotels serviced by robots, to the best of our knowledge there are no studies of direct experiences that analyse the five customer personalities. Second, we reveal important aspects of the process that drives experience appraisal, highlighting efficiency and stimulation as the main drivers of attitude. Third, we show how the more or less marked of the big five personality traits moderate the assessment of the experience undergone, as well as the drivers of the attitude towards service provision by a social robot.

## Conceptual Framework

Although the literature addresses several lines of research on the adaptation of robots in service organisations (mechanoids, chatbots, humanoids and androids), only humanoid robots equipped with social intelligence protocols will be considered in this study. Social intelligence protocols allow technology-mediated services to provide customer interactions with adequate support and assistance [24], that is, with the essential elements to deliver a front-office service [25]. For example, Aymerich-Franch and Ferrer [3] classified tasks performed by social robots into three categories: First, intermediation tasks to reduce human–human interaction, such as patient reception (patient registration, check-in/out, providing information about patients, assigning patients to care units, etc.) [26]. Second, assisting with communication and monitoring tasks, such as reminding patients to take prescribed medication, managing doctors' appointments, reminding visitors of behaviour patterns in public places, detecting people without a mask or scanning the temperature of visitors at the entrance to the establishment, among others. Third, improving patients’ well-being, such as providing a conversation, offering advice on maintaining healthy habits, dancing, singing or facilitating Internet browsing, among others [3].

Practically all activities collected by Aymerich-Franch and Ferrer [3] could be framed within front-office services, since, unlike back-room services, they are characterised by a high degree of contact between service providers and customers [10]. Furthermore, all contact is facilitated by exchanging words, for example, by greeting guests [27], providing advice and information while completing a task [20, 28] or in the form of feedback when the task has been completed [20, 29]. Therefore, a basic feature of front-office service robots is their ability to establish a conversation with users, where they can transmit emotional support signals, including messages of pity and sorrow for people in aversive situations, and of happiness and pride for people in favourable situations [25, 30]. These skills are developed and provided by social intelligence protocols.

### Social Intelligence Protocols in Social Robotics

Although there is a growing body of service management literature on the use and implementation of social robots in service organisations [10], the study of protocols to produce social intelligence is basically confined to the field of social robotics [15, 17, 18].

Remarkable progress has recently been made in robot communication protocols by reproducing a more natural language, although it is not clear whether it is as effective as human-to-human communication or in which contexts it would be more practical to use it [31]. In this sense, Anzalone et al. [32] proposed the concept of humanoid “intelligence” to refer to the human perception that robots act rationally, that is, as if they had cognitive abilities. They further explained that this perception only occurs when all AI algorithms act together in a coordinated manner during interaction with humans. Therefore, when it is said that a robot has social intelligence, the perception of humans that the robot has social skills is considered, which would be the result of the joint action of communication protocols [20] and would include the expression of emotions, the ability to negotiate, persuade, explain their behaviour and provide emotional support [33]. In fact, emotional support contributes to reducing the stress that may be generated by the situation and improves the valuation of services, as well as the persistence of clients in solving problems [25, 34].

A communication protocol is a set of rules and/or procedures that allow two systems to communicate with each other, whether technological or human [35]. Thus, the communication protocol defines the rules and principles that govern the exchange of information between two systems, which includes syntax (the combination of words to build phrases and sentences), semantics (the interpretation or meaning of words and phrases), communication timing, and possible error recovery methods [36]. However, although thanks to the use of algorithms and data structures, communication protocols between digital systems have been widely developed [36], communication between humans and robots is more complex due to the numerous elements of verbal and non-verbal signals that humans use [20] and their ability to convey emotions with language [25]. For the implementation of communication protocols using language, three elements must be considered: what is said, how it is said, and to whom it is said [37]. In social robots, what is said is programmed into the script, while how it is said is managed by various modules of transmission protocols [36]. For example, a message delivered by a verbal language module (via text-to-speech programmes) can increase its persuasiveness if combined with non-verbal expression modules (with gesticulations, changes in gaze or by regulating the tone of voice towards a higher or lower pitch) [38]. Thus, in an experiment to estimate the persuasiveness of verbal and non-verbal cue modules, the results showed that while non-verbal manipulations significantly improved people's response to the robot's suggestions, verbal cue manipulations did not [38]. Furthermore, coordination between the script, with expressions of empathy and concern [39], and the verbal and non-verbal cue modules may be able to convey emotions capable of enhancing the mental state, the affiliative feelings and the reassurance of the recipient [25].

In terms of to whom it is said, Jost et al. [40] proposed the concept of "understanding" to refer to the degree of effectiveness achieved by verbal and non-verbal protocols in social communication. To implement social intelligence protocols in service robots, managers of service organisations need to follow the cooperative principle [41]. In other words, they must have clear expectations about their target audience's prior knowledge of what the robot is going to explain to them and their ability to understand it [41, 42]. Therefore, knowing the degree of motivation and the cognitive abilities of the recipients is relevant to interpret the effectiveness of social communication protocols, and one of those key variables that predispose customers to social interaction is personality.

### The Experience as a Result of the Provision of Service

In the literature on service management, the service encounter is considered the "moment of truth", due to the critical value that this experience has in building the perceived quality of the service [31]. The outcome of providing a service to customers is called the experience [11, 31, 43] and in the co-creation of this experience, the supplier-customer interaction is essential [31]. Therefore, in a service delivered by a social robot, the robot will be the key agent for co-creating experiences [44].

The experience is a holistic construct, in the sense that it incorporates cognitive, emotional, sensory, social and spiritual responses to all customer-company interactions [43]. In other words, the experience is not a snapshot of a specific moment, but rather it gathers the experiences accumulated during the three phases of service delivery, namely: pre-service encounter, which covers the information received before use; service encounter, which continues during use; and post-service encounter, which includes feedback and assessments after use [31]. As a result of this accumulated experience, users may maintain or change their attitude towards the service delivered and therefore modify their intention to continue using that service [41, 43, 45]. Attitude is a mental construct of an emotional nature that reflects the positive or negative affection towards an object or service received, as a diagnosis of the experience and previous experiences [41, 45].

To support the causal relationship between attitude and intention to use, the theory of reasoned action of Ajzen and Fishbein [46] has been considered. According to these authors, attitude (an intrinsic psychological construct of the user) and subjective norms (defined as the perceived social pressure) are precedents of the behavioural intention. However, in this study only the attitude and not the subjective norms have been considered, since the experience was acquired individually and was valued just after undergoing it, and without time to receive social pressure, that is, without being able to talk about it with family, friends or acquaintances. Although Ajzen and Fishbein [46] considered that attitude was an intrinsic variable and therefore unobservable, they also thought that it could be shaped by the experience of interacting with the environment, that is, by external factors. Hence, the experience of receiving the provision of a service by a social robot will contribute to shaping the consumer's attitude towards the robotic agent that delivers the service [44]. This study proposes that, if a customer evaluates for the first time a service delivered by a social robot equipped with social intelligence protocols, the evaluation of this experience will contribute to changing her/his attitude. In addition, if it is positive, it will also contribute to a greater behavioural intention, in the sense of a greater predisposition to continue receiving such service. Based on the above, the following hypothesis is proposed.

- **H1**An increase in the favourable attitude towards the service delivered by a social robot will increase the intention to continue receiving services delivered by robotic equipment.

Front-office services delivered by social robots also generate holistic experiences [31], in the sense that they incorporate cognitive responses, consisting of functional delivery (such as problem solving), socio-emotional responses (such as expressions of empathy and concern) and sensory-spiritual responses (derived from the relational link of the HRI) [10, 31, 43, 47].

Previous research, for example, Rauschenberger et al. [48], already proposed that the user experience was a combination of aspects related to efficiency and effectiveness (of a cognitive-functional nature) together with aspects related to aesthetics, pleasure of use or attractiveness (of an emotional, sensory and spiritual nature). Along this same line of argument, in their sRAM model, Wirtz et al. [10] proposed the existence of a link between three factors: one linked to the characteristics of efficiency and effectiveness in the functional provision of the service, another to the aesthetic characteristics, generators of pleasure and attractiveness, and, finally, with socio-emotional and relational elements. However, the characteristics linked to the factors that generate a social response have not been considered. Although initially this could be considered a limitation of the proposal by Rauschenberger et al. [48], in the case of first experiences, where social approval has not yet been received, this factor is not decisive [41].

Thus, in the first group of criteria, of a cognitive-functional nature, Perspicuity, Efficiency and Dependability are considered [48]. Perspicuity refers to the perceived degree of ease or difficulty of understanding how the service provided by the robot works. Evidence has been collected showing that the use of social robots in the provision of services can contribute to facilitating and improving the customer experience [44, 49]. On the other hand, efficiency is the ability of the social robot to solve the customer's problem in the shortest possible time and/or with the minimum resources possible. Robots have the ability to provide services accurately, reliably, efficiently, conveniently and quickly [10]. Finally, dependability refers to the degree of consistency and perceived stability of the services provided by the social robot. When comparing the benefits of social robots with self-service technologies (e.g. ATM), the unstructured interface of robots stands out, allowing them, for example, to guide the customer through a scripted process (complete a transfer through an ATM) and can even help them correct the mistakes they may make, which makes the service delivered much more robust than that of an ATM. That is, in this service provision environment with a simple emotional-social load, the robot could act as if it were a service employee) [8, 10]. Based on these definitions, the following hypotheses are proposed:

- **H2** A positive assessment of the perceived perspicuity of the service provided by the social robot will positively influence the consumer's attitude.

- **H3** A positive assessment of the perceived efficiency of the service provided by the social robot will positively influence the consumer's attitude.

- **H4** A positive assessment of the perceived dependability of the service provided by the social robot will positively influence the consumer's attitude.

However, front-office service provision must not only provide the core of the service, but also its social-emotional and relational elements [50]. In this sense, Rauschenberger et al. [48] proposed two factors that could explain the hedonic experience: Stimulation and Novelty. Stimulation refers to the emotional incentives derived from the provision of the service performed by the social robot. Social robots with social intelligence protocols that allow them to communicate with humans through verbal and non-verbal language contribute to improving the relationship, making it more valuable [51]. Furthermore, when robots engage in collaborative tasks with users, this cooperation stimulates engagement with the service provider [52]. Also, Van Pinxteren et al. [53] noted that robots with an anthropomorphic design arouse greater confidence in users and, at the same time, increase the perception of enjoyment. Novelty, on the other hand, refers to the perception that the provision of the service by a social robot is something innovative or creative that can encourage its use. Chandralal and Valenzuela [54] highlighted that, in a travel context, first experiences in environments totally different from the usual ones, had a significant effect on the evaluation of the experience. Similarly, a first experience of service delivery by a social robot can be expected to increase the perceived novelty of the service. Based on these definitions, the following hypotheses are proposed:

- **H5.** A positive assessment of the stimulation perceived by the service provided by the social robot will positively influence the consumer's attitude.

- **H6.** A positive assessment of the perceived novelty of the service provided by the social robot will positively influence the consumer's attitude.

### The Personality of Consumers in the Evaluation of the Service Experience with Social Robots

Given the distinctive peculiarity of front-office service delivery, where socio-emotional drivers often play a more important role than those of a cognitive-functional nature, individual customer characteristics, such as gender, age, social class, demographic data, personality traits, etc., often play a determining role in adjusting the operational design of the service [55]. If the service provider is a social robot, it is also important to determine the specific design characteristics that will generate memorable experiences and, perhaps even more importantly, for long-term experiences [31]. In other words, to deliver memorable experiences it is necessary to offer personalised services, which, in the case of using social robots, means that the robot will have to learn and adapt to the individual’s tastes [56] or personality [16, 18]. Although several schools of thought have attempted to explain personality theories, this study is conducted from evolutionary psychology, according to which personality is a neurological or biological mechanism that humans have developed for evolutionary purposes [57, 58]. For example, Figueredo et al. [58] defined personality traits as the result of ontogenetic variations of a random nature that occur during the embryonic period of individual development and, therefore, albeit with constant changes and adaptations, they are relatively stable throughout life. However, although each school of thought has different views of the mechanisms through which personality is formed and expressed, they all consider it a predictor of human behaviour [16].

The study of customers’ personality traits is important since they are relatively stable over time, and even continue to hold when situations change [59–61]. This stability motivated their use in social robotics from very early on [16]. Hence, numerous studies have assumed that human personality might play a moderating role, in the sense that it determines whether an individual would be more or less likely to interact with a robot and whether those interactions would be pleasant [18].

However, despite the fact that there is currently some consensus that personality can be characterized by traits and that these make up the big five personality factors proposed by McCrae and Costa [21], reaching this point has not been free of controversy [62]. Since the traits emerged through statistical analysis (factor analysis), the debate was opened on whether personality traits were actually something constructed by researchers and, therefore, did not really exist. To answer this question, Figueredo et al. [58] have shown that personality exists as a definable construct that characterises the individual and, therefore, it is not something constructed by the observer.

The big five traits proposed by McCrae and Costa [21] are: extraversion, agreeableness, conscientiousness, neuroticism and openness to new experiences. They characterised each of them with two opposite poles (high pole and low pole). However, different degrees of sharpness between them still persist, so while extraversion, neuroticism and conscientiousness can be recognised more clearly, the traits of agreeableness and openness to new experiences are somewhat vaguer and remain more open to interpretation. In social robotics too, the Big Five model has been the predominant one in HRI studies [17, 18].

The extraversion trait is related to sensitivity towards obtaining rewards, and is also associated with the quest for greater social affiliation and/or the achievement of greater social status [63, 64]. It is one of the traits that is most considered as a moderator in HRI, since the most extroverted people tend to be more willing to interact with robots [16–18], more likely to talk to them [65], and more trusting of them, compared to less extroverted ones [66]. In addition, extroverts report greater positive attitude change after HRI experiences than introverts [67]. However, in an experiment in a backroom office context whereby a robot is tasked with reminding employees of their work schedules, the results indicated that it was the less extroverted workers who were more motivated by the robot to finish their work quicker than the more extroverted ones [68].

The neuroticism trait is characterised by the tendency that some individuals experience towards an increase in negative emotions, derived from a greater sensitivity to threats and danger of punishment [64]. This is the second most studied trait as a moderator in HRI [16], since it often plays an antagonistic role [18]. For example, Damholdt et al. [67], who estimated the change in attitude of a sample of elderly people after an HRI experience with a teleoperated robot, found that more neurotic profiles tended to be the ones who viewed robots as less human. Furthermore, in a study on human–robot proxemics, meaning the personal space that people create between each other, they found that more neurotic people put a greater distance between themselves and robots than less neurotic ones do [69]. Conversely, the study by Cruz-Maya and Tapus [70], which proposes a scenario where a robot or tablet is the medium used to teach a multimedia course on nutrition and healthy eating, showed that participants with a high level of neuroticism scored better in the test than the less neurotic ones.

The conscientiousness trait is defined by maintaining a stable behavioural pattern, which implies directing actions towards achieving goals and delaying gratifications. In addition, it is considered an accurate trait in health forecasts, since it can be used to predict longevity, the onset of certain diseases, and health-related behaviours [71]. In the case of HRI, it is one of the least analysed profiles as a moderator and, moreover, the most neutral in its effect on the intention to use social robots [16]. In their meta-analysis, Esterwood et al. [16] found no evidence that the degree of conscientiousness affected the willingness to accept social robots. However, some evidence has also been collected that assigns a more active role to conscientiousness. For example, in their aforementioned backroom office scenario, Cruz-Maya and Tapus [68], showed that highly conscientious people were more likely to obey the robot's instructions about the schedule, and completed the task quicker than people with low conscientiousness, i.e., their profile may be a good predictor of better task performance. In addition, more conscientious people tend to perceive robots as more able to adapt to their needs, including social needs, so they are more willing to use them than those who are less conscientious [72].

Regarding the less precise traits, agreeableness is linked to predispositions to altruism and empathy, the ability to understand the emotions of others [73]. This is another of the least used profiles as a moderator in HRIs, despite being a user profile that expresses a positive acceptance of social robots [16]. For example, in an experiment recreating a smart laboratory apartment served by a robot, the most agreeable users rated the experience more positively than the less agreeable ones [74]. Takayama and Pantofaru [69] in a proxemics experiment, found that participants who expressed a high degree of agreeableness stayed closer to the robots than those who expressed a low degree of agreeableness.

Finally, openness to new experiences is a profile characterised by a brilliant imagination and an interest in intellectual issues [73]. Although this is a profile that has been used discreetly as a moderator in HRI, it is a profile that manifests a positive acceptance of social robots [16]. For example, in scenarios where robots are used as teaching assistants, it was the more open teachers who expressed greater acceptance of these robots, and greater belief that their use would improve their daily activities [72]. The results of another study, involving an android robot advertising a Bluetooth headset, showed that participants with a more open profile, may have deemed the artificial agent unfriendly and extroverted, but expressed a greater willingness to spend money on the advertised product [75].

This study proposes to explore how consumers’ personality traits affect the evaluation of the experience of receiving a service from a social robot, and the following theoretical precedents have been considered to explain the attitudinal change. The first is media equation theory, i.e. the tendency for people to equate robots with real social actors. Previous research has assigned human and personality attributes to digital systems (Apps, chatbots) that manifest human skills [7, 25]. For example, Dryer et al. [19] conducted several studies to explore the relationships between humans and artificial agents, and the results showed that people perceived the personalities of artificial agents according to their same dimensions of human personality. Thus, they rated artificial agents as calm, organised, curious, competitive, withdrawn, anxious, lax or closed-minded, among other attributes [19]. In the case of social robots, it has been argued that their mere appearance and behaviour already induces people to consider them as having their own identity and personality [12, 13]. Second, the cognitive theory of consensus bias proposes that people have a tendency to believe that other people have the same beliefs, convictions and evaluations of reality, that is, to consider that others are like them [41, 76]. Third, the similarity attraction hypothesis, according to which people are more attracted to, and prefer to interact with, those people who are demographically, ethnically, politically, and personality-wise similar to them [14, 77]. Fourth, the complementarity attraction hypothesis which, on the contrary, holds that people are attracted to others whose personality characteristics are complementary to their own, so that their own personalities, especially the dominance/submission dimension, can be balanced [78, 79].

Both the human–computer interaction and HRI literature have supported the similarity attraction hypothesis [80, 81]. For example, Nass and Lee [80] in experiments with Computer-Synthesized Speech found that people exhibit similar attraction to computer-generated speech, even when personality was clearly not relevant. Along the same line of argument, in HRI it has been found that humans prefer robots that have a personality similar to their own, i.e. extroverted people prefer extroverted robots and introverted people prefer introverted ones [82, 83]. However, evidence to the contrary has also been collected. Woods et al. [76] found that participants in an HRI experiment rated themselves as having stronger personality traits than the robot. Furthermore, in the case of interactions with on-screen computer characters, results showed that participants tend to prefer a character whose personality is complementary, rather than similar, to their own [78]. Analogous results have also been collected in HRI, describing experiences in which participants enjoyed interacting with a robot with a personality complementary to their own, and whom they considered more intelligent, more engaging and more socially present than a robot with a similar personality [15].

Therefore, it is plausible to consider that each person will feel that the social robot s/he interacts with while receiving the service will have the same personality trait [57], that is, s/he will assume that the robot is like her/him, and, however, depending on her/his trait activation (similarity/complementarity), s/he will value the experience differently to another person.

## Methodology

In order to test and validate the hypotheses proposed regarding the evaluation of the experience, and to explore how customers’ personality traits contribute to moderating the assessment of this experience, an experiment was proposed to test a prototype service delivered by a social robot equipped with an AI system and social intelligence response protocols. A stand was set up on the campus of the University of Barcelona, where a large number of people pass by every day, in order to collect a large sample in a week.

### Experiment and Scales

**Fig. 1:**
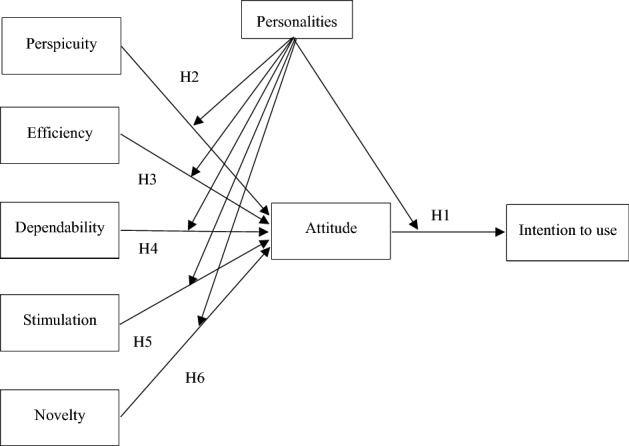
Proposed model. *Note*: The graph shows how the five components of experience explain attitude and, in turn, usage intention. Personality traits also moderate these effects

To recreate front-office service delivery, a service prototype was designed, as is common practice to test the service experience prior to launch [84]. Service prototypes, which can be real or virtual, capture in the form of models the essential characteristics of the service to be implemented in order to explore how the different tangible and intangible aspects work, as well as the reactions of different stakeholders [85, 86]. Such prototypes that simulate service experiences [84] are used when real applications may be too complicated, time-consuming or expensive to carry out [87]. Furthermore, as Wolfe and Roberts [88] pointed out, their results are similar to those obtained with real field experiments (Fig. 1).

**Fig. 2:**
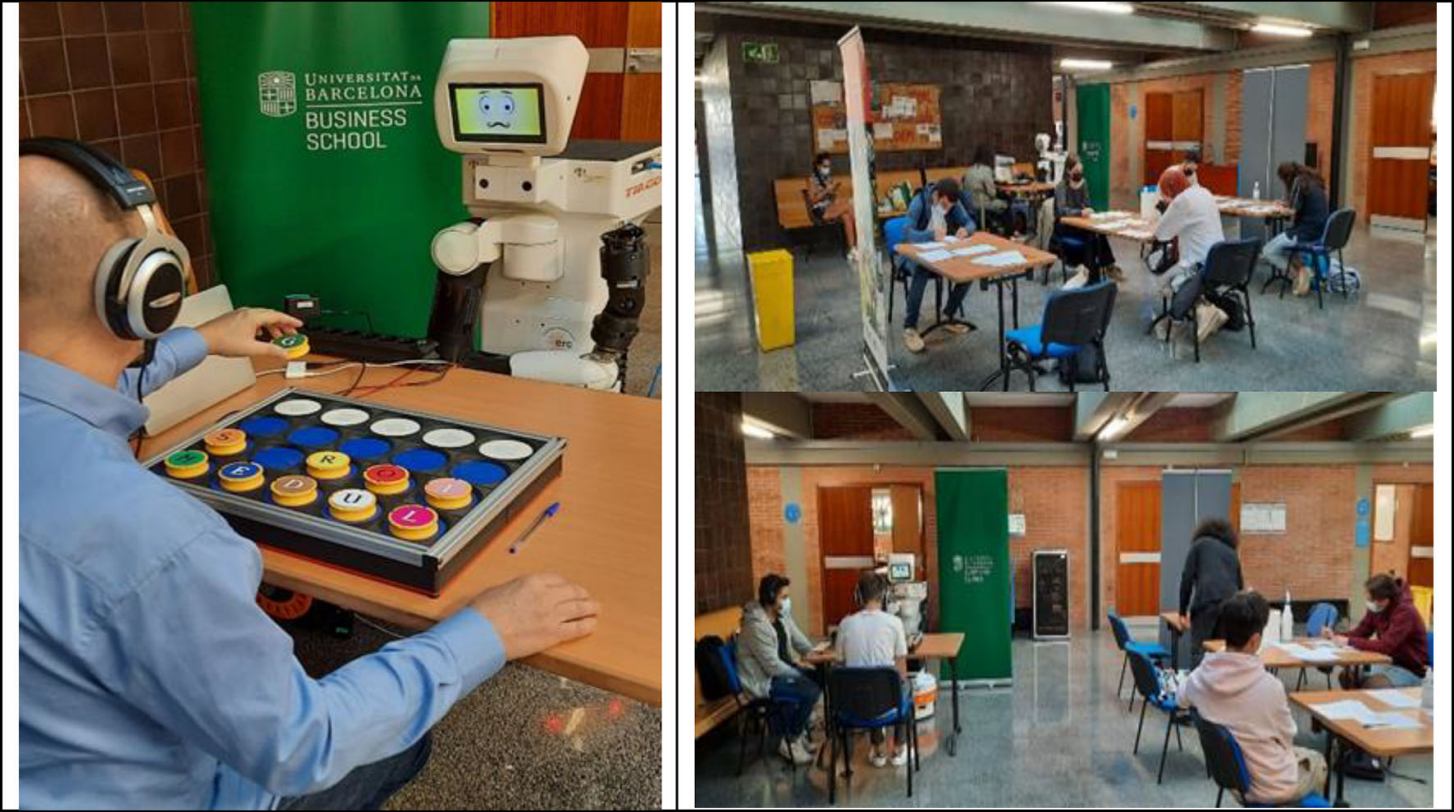
On the left, a participant playing the game with the help of the robot and, on the right, two general images of the scenario

A board game is proposed as a prototype that reproduces in a behavioural model the essential elements of the service in terms of time, assistance requirements and robot attention [84]. The board game consisted of forming the five-letter name of a Nobel Prize winner (e.g. "MORSE") from ten letters available in the form of tokens (Fig. 2 shows an image of the game) [45]. Thus, the game captures: (1) the duration of the experience (about five minutes), which is very similar to that of a hotel check-in [89]; (2) a sequence of steps with a risk of getting stuck as is common in complex ATM transactions [8]; (3) the provision of help by the robot, through hints and suggestions, messages of empathy and reassurance in adverse situations and congratulations in favourable situations, which are all common activities in customer services [28].

In addition, to measure experience, although this concept has been discussed for more than two decades in the literature, no consolidated instruments are yet available [10, 31, 43, 47]. For example, one of the latest attempts to construct a measurement instrument has been the proposal by Luo, Lam and Fan [90], who considered the use of seven constructs to measure the past experience of an entertainment tourism service. Although they used a construct linked to cognitive responses and several constructs linked to emotional, sensory and spiritual responses, they did not have any constructs related to functional performance or social acceptance, the latter being important when assessing past experiences. In short, there is still research to be carried out in order to obtain consolidated instruments for measuring a service experience.

This study used a scale called the User Experience Questionnaire proposed by Rauschenberger et al. [48], which was developed in an interactive product evaluation environment and is made up of 20 semantic differentials (each starting with either the positive statement or the antonym) that made up five experience evaluation constructs: Perspicuity, Efficiency, Dependability, Stimulation and Novelty. The Spanish translations of the User Experience of Interactive Products scales developed and validated by Rauschenberger et al. [48] were used. They were presented in the form of a semantic differential and were to be evaluated using a seven-point Likert scale (1 = "strongly disagree" and 7 = "strongly agree"). In addition, two scales commonly used in the literature on social robotics, attitude (consisting of three items) and intention to use the social robot (consisting of three items) from Forgas-Coll et al. [20], were used as dependent variables. In this case, the items were to be evaluated using a five-point Likert scale (1 = "strongly disagree" and 5 = "strongly agree"). Both the semantic differentials and the items of the attitude and intention to use scales are described in Table 2.

Regarding personality measures, there are numerous validated scales to measure the five characteristic traits, such as the 1985 NEO Personality Inventory, which had 240 items, or its 1992 revision, with 60 items [73]. However, they are so long that their use in HRI is almost prohibitive, so most studies have used personality inventories consisting of ten items to reduce the time participants have to spend answering the questions [91]. Similarly, the one proposed in this research, the BFI-10 scale, consists of eleven statements [92]. The questions in the questionnaire begin with "I see myself as someone who…" and eleven continuations are proposed: “… is reserved (R), … is generally trusting, … tends to be lazy (R), … is relaxed, handles stress well (R), … has few artistic interests (R), … is outgoing, sociable, … tends to find fault with others (R), … does a thorough job, … gets nervous easily, … has an active imagination, … is considerate and kind to almost everyone". All of these were rated on a five-point Likert scale (1 = "strongly disagree" and 5 = "strongly agree") and (R) indicates items that were reverse-scored [93].

### Robotic Equipment

The robot used for the service deliver was a TIAGo, a highly versatile semi-humanoid robot that combines perception, navigation and AI manipulation skills. It also has one of the fastest and most efficient processors on the market (NVIDIA® Jetson™ TX2), which allows functional activities to be programmed, as well as social communication protocols and the coordination of social functions [94]. Control of the robot's interactions with the participants can be Wizard of Oz, pre-programmed or automation autonomy [18]. The difference between the three is that while Wizard of Oz presents the robot as apparently autonomous, it is actually controlled remotely by a human operator. In the pre-programmed strategy, the apparent autonomy is represented by triggering predefined responses that are the same for all interlocutors, regardless of the human's behaviour. Finally, automation autonomy is based on the robot itself reacting independently to the human, and depending on the human's response. In this study, although the basis of the argument is pre-programmed, there is variation in response depending on whether the participant moves the right or wrong token.

To adapt the robot to the delivery of the service, three algorithms were programmed: an algorithm to collect all the operational information coming from the board game, so the electronic board was connected to the robot’s operating system, where any movement of the tokens was registered and recorded, as well as all the derived information (time the player keeps the tile, if s/he took the correct tile, etc.). A second processing and response algorithm activated the timer that started the response subsystem, as the first movement was initiated. This subsystem consisted of a message that provided hints and clues on where to find the correct tokens (functional support) and, depending on whether the move was correct or incorrect, messages were issued as expressions of empathy and concern (emotional support) [93]. Finally, a social intelligence protocol transformed the script into verbal and facial cues, which conveyed the impression that the robot was having a conversation, similar to the one used in chatbots by Dryer [19]. To reproduce the verbal signals, Loquendo text-to-speech software (which transformed the text of the script into sound in Spanish) was used. The script (structure of messages to be reproduced) had three parts: (1) introductory messages when activating the game, where the robot introduced the game and explained to the player the type of help it was going to offer; (2) in-game messages, the system counted the time it took to move a token and gave advice on where to look (why don't you look in the centre?), along with messages with an emotional content. When the player picked up the wrong token, the robot emitted discouraging messages such as "Mmmmh", "Really?" and "Are you sure?", whereas if the player took the correct token, the messages were encouraging, such as "Great", "Yes" and "Wow"; and finally, (3) a farewell message, when the participant finished the game. To increase the convincing power, verbal language was accompanied by non-verbal language. To generate it, graphic design software was used to recreate facial expressions in cartoon form (an example of these facial expressions is shown in Fig. 2).

The joint and simultaneous action of the three subsystems (collection, processing and response) generated the perception that the TIAGo robot was acting intelligently and making autonomous decisions [95].

### Participants and Procedure

During the week that the stand was installed on the campus of the University of Barcelona, 378 participants were recruited (Table 1 shows the demographic data of the participants). All of them were volunteers and received no compensation and, after signing their consent, tried to complete the name of the Nobel laureate. Thanks to the help of the TIAGo robot, they all managed to complete the game in less than five minutes. Once the game was over, they completed a questionnaire consisting of items rating experience, attitude and intention to use, together with items from the five-factor personality model. Finally, participants were asked to fill in their identification data.

**Table 1:** Demographic profile of the respondents

Variable	Description	Frequency	Percentage
Gender	Male	188	49.7
	Female	190	50.3
Age	18–24 years	233	61.6
	25–34 years	61	16.1
	35–44 years	24	6.4
	45–54 years	29	7.7
	More than 54 years	31	8.2
Nationality	Spanish	309	81.7
	Rest of Europe	15	4
	North American	2	0.5
	South American	26	6.9
	Asian	20	5.3
	Others	6	1.6

Once the questionnaires had been collected, the items and ratings were recoded with reverse-scoring, and then the experience rating model (shown in Fig. 1) was validated using structural equations (SEM) based on variance and covariance matrices by maximum likelihood with EQS 6.4 [96]. Subsequently, to explore the moderating effect of personality, ten models (two models, for the two extreme poles of each personality profile) were estimated using OLS.

## Results

### Validation of the Scales

Once the data had been collected, the psychometric characteristics of each item were analysed with respect to its scale (latent variables), confirming the 26 items that make up the seven scales. Table 2 describes the weight for each item (its correlation with respect to its scale), the composite reliability (CR), the convergent validity of the scales used (AVE) and, in addition, a Cronbach's α coefficient as an index of the reliability of the scales. Each factor loading exceeded 0.6 and the t-values for each item were significantly high as recommended by the literature [96]. All constructs achieved a Cronbach's α of around 0.80. Composite reliability (CR) also remained around 0.80 (ranging from 0.62 for dependability to 0.92 for stimulation) and all items showed adequate convergent validity. Furthermore, Table 3 shows the discriminant validity of the scales, where the square root of the AVE of each scale is higher than the correlations with the rest of the scales, i.e. none of the values below the diagonal of the matrix reach the values of the diagonal [97].

**Table 2:** Analysis of the dimensionality, reliability and validity of the scales (mean and SD)

	Factor loading	T	Mean	SD
*Perspicuity (AVE: 0.58; CR: 0.80; C. Alpha: 0.79)*				
not understandable/understandable	0.76***	15.90	1.00	1.51
easy to learn/difficult to learn (R)	0.66***	11.03	1.57	1.92
complicated/easy	0.69***	13.05	0.75	1.80
clear/confusing (R)	0.71***	13.20	1.04	1.80
*Efficiency (AVE: 0.68; CR: 0.87; C. Alpha: 0.86)*				
fast/slow (R)	0.78***	15.05	1.44	1.42
inefficient/efficient	0.82***	18.00	1.41	1.45
impractical/practical	0.78***	15.91	1.63	1.4
organised/cluttered (R)	0.78***	18.02	0.92	1.6
*Dependability (AVE: 0.62; CR: 0.62; C. Alpha: 0.82)*				
unpredictable/predictable	0.78***	17.61	0.35	1.66
obstructive/supportive	0.63***	10.92	1.37	1.59
secure/not secure (R)	0.76***	16.73	0.77	1.75
meets expectations/does not meet expectations (R)	0.78***	17.02	0.81	1.72
*Stimulation (AVE: 0.77; CR: 0.92; C. Alpha: 0.91)*				
valuable/inferior (R)	0.88***	21.59	1.01	1.50
boring/exciting	0.82***	18.98	0.72	1.49
not interesting/interesting	0.88***	20.44	1.29	1.51
motivating/demotivating (R)	0.85***	20.41	0.96	1.52
*Novelty (AVE: 0.74; CR: 0.90; C. Alpha: 0.90)*				
creative/dull (R)	0.82***	18.79	1.23	1.57
inventive/conventional (R)	0.86***	22.96	1.17	1.59
usual/leading edge	0.81***	17.65	1.18	1.54
conservative/innovative	0.86***	24.52	1.21	1.62
*Attitude (AVE: 0.72; CR: 0.86; C. Alpha: 0.86)*				
I think it is a good idea to use the robot	0.81***	14.37	3.66	0.94
For me, the robot is interesting	0.84***	15.73	3.93	0.98
I consider it correct to use the robot	0.82***	17.87	3.78	0.99
*Intention to use (AVE: 0.62; CR: 0.79; C. Alpha: 0.79)*				
If the robot was available, I would try to use it	0.79***	15,50	3.40	1.13
If the robot was available, I would try to use it whenever I could in my spare time	0.80***	15,28	2.77	1.21
If the robot was available, I would sometimes think about when I could use it	0.64***	10,10	2.24	1.12

The model fits Chi-square (χ^2^): 269.3825; df: 252; p: 0.21573; RMSEA: 0.014; CFI: 0.997; NNFI: 0.996

*AVE* average variance extracted, *CR* composite reliability

*p < 0.05; **p < 0.01; ***p < 0.001; (R) Item is reverse-scored

**Table 3:** Discriminant validity of the scales

	Perspicuity	Efficiency	Dependability	Stimulation	Novelty	Attitude	Intention to use
Perspicuity	0.76						
Efficiency	0.44***	0.82					
Dependability	0.07 (ns)	0.18**	0.79				
Stimulation	0.48***	0.40***	0.17**	0.88			
Novelty	0.19**	0.28***	0.09 (ns)	0.44***	0.86		
Attitude	0.38***	0.46***	0.14*	0.47***	0.29***	0.85	
Intention to use	0.31***	0.37***	-0.05 (ns)	0.50***	0.15*	0.66***	0.79

Below the diagonal: correlation estimated between the factors

Diagonal: square root of AVE

*p < 0.05; **p < 0.01; ***p < 0.001

### Model Analysis

The SEM-fitted intention-to-use model achieved acceptable *R*^2^ values for the sample size used [98]: *R*^2^ = 0.29 for attitude and *R*^2^ = 0.47 for intention to use (see Table 4).

**Table 4:** Causal relations in the general model

Independent variable	Dependent variable	Beta	T	R^2^
Perspicuity	Attitude	0.135*	2.00	0.297
Efficiency		0.262*	3.65	
Dependability		0.015	0.24	
Stimulation		0.225*	2.74	
Novelty		0.135*	1.98	
Attitude	Intention to use	0.689*	10.49	0.474

Significant at *p < 0.05

**Fig. 3:**
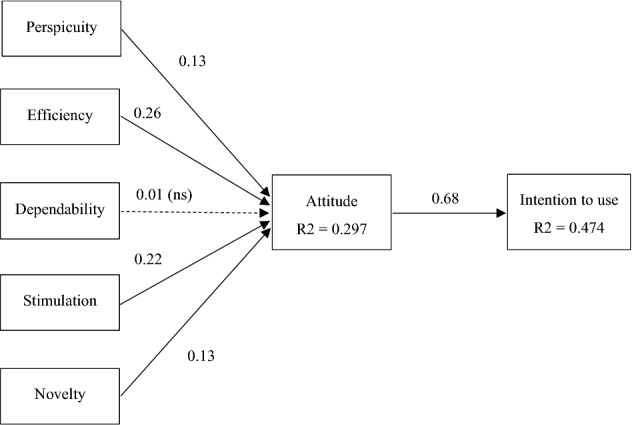
General Structural Model Results (*p* < 0.05)

From Table 4 and Fig. 3 it can be seen that the Intention to Use the service provided by a robot equipped with an AI system is highly correlated with Attitude (β = 0.68, *p* < 0.05), which indicates that it is in agreement with the usual results in the TAM literature [44, 99], and supports H1. Four of the five hypotheses posed that explain how the experience with the social robot contributes to improve the consumer's attitude have reached significant values and with the sign predicted in the hypotheses. Thus, among the functional elements, efficiency, defined as the ability of the social robot to solve the customer's problem in the shortest time and with the fewest resources is the one that has achieved the greatest weight (*β* = 0.26, *p* < 0.05), followed by perspicuity, which refers to the ease of understanding how the service provided by the robot works, (*β* = 0.13, *p* < 0.05). However, the degree of Dependability, which refers to the degree of consistency and perceived stability of the services provided by the social robot, does not reach a significant value. In short, there is evidence to support H2 and H3, but not H4.

As for the factors related to socio-emotional and relational elements, stimulation, which refers to the emotional incentives derived from the provision of the service by the social robot, was the factor with the highest weight (*β* = 0.22, *p* < 0.05). It was followed by novelty, which refers to the perception that the provision of the service by a social robot is something innovative or creative, whose weight was *β* = 0.13, *p* < 0.05. In short, there is evidence to support H5 and H6.

Given that five of the six hypotheses have been confirmed and with the same sign as the one predicted, it can be considered a valid model to explain the consumer's experience after an interaction with a social robot.

**Fig. 4:**
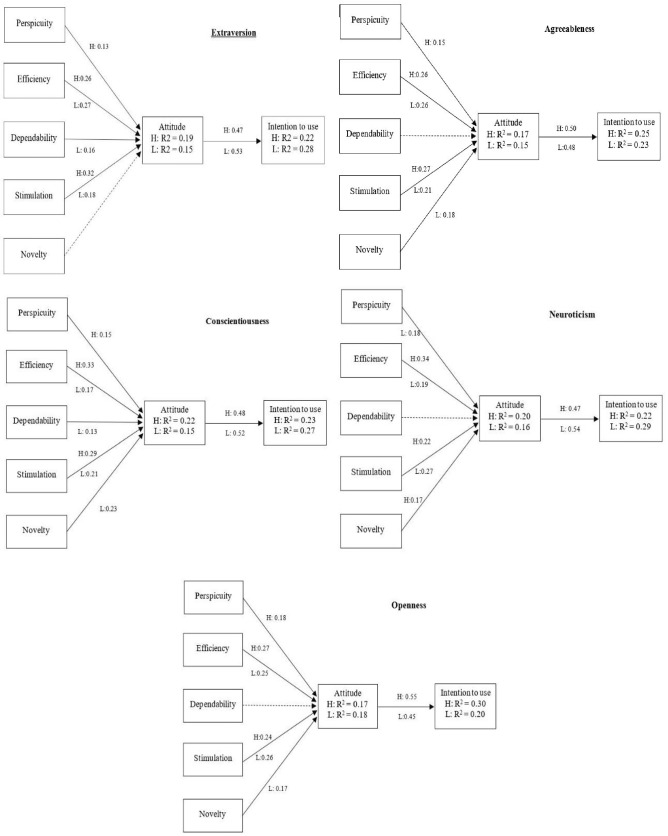
Causal relationships of the 5 personality factors. Only the significant values are shown (*p* < 0.05). *H* High, *L* Low

To explore how consumers' personality traits rated their experience with the social robot, this study took the self-completed values of the BFI-10 scale [92]. Based on the scores obtained, the sample was divided according to each of the five personality traits (Extraversion, Agreeableness, Conscientiousness, Neuroticism, Openness). For each personality trait, the two extreme poles were considered, those that reached values above the mean, which were labelled as High, and those that reached values below the mean, which were labelled as Low. For each of the subsamples, the experience rating model was estimated using OLS (Tables 5, 6, 7, 8, 9 and Fig. 4 show the results obtained).

**Table 5:** Causal relations extraversion factor

Independent variable	Dependent variable	Low			High		
		Beta	T	R^2^	Beta	T	R^2^
Perspicuity	Attitude	0.076	1.09	0.150	0.130*	1.97	0.197
Efficiency		0.279*	3.73		0.263*	4.31	
Dependability		0.160*	2.25		0.000	0.00	
Stimulation		0.186*	2.06		0.324*	4.80	
Novelty		0.082	1.06		0.078	1.20	
Attitude	Intention to use	0.537*	7.81	0.289	0.474*	7.19	0.224

Significant at *p < 0.05

**Table 6:** Causal relations agreeableness factor

Independent variable	Dependent variable	Low			High		
		Beta	T	R^2^	Beta	T	R^2^
Perspicuity	Attitude	0.063	0.84	0.154	0.153*	2.44	0.172
Efficiency		0.260*	3.91		0.265*	3.85	
Dependability		0.041	0.55		0.046	0.78	
Stimulation		0.219*	2.50		0.276*	4.22	
Novelty		0.181*	2.17		0.013	0.23	
Attitude	Intention to use	0.489*	7.45	0.239	0.503*	7.18	0.253

Significant at *p < 0.05

**Table 7:** Causal relations conscientiousness factor

Independent variable	Dependent variable	Low			High		
		Beta	T	R^2^	Beta	T	R^2^
Perspicuity	Attitude	0.045	0.63	0.154	0.152*	2.46	0.228
Efficiency		0.178*	2.65		0.335*	5.11	
Dependability		0.133*	1.82		− 0.037	− 0.68	
Stimulation		0.215*	2.85		0.298*	4.10	
Novelty		0.237*	3.12		− 0.056	− 0.94	
Attitude	Intention to use	0.522*	8.03	0.272	0.482*	7.07	0.233

Significant at *p < 0.05

**Table 8:** Causal relations neuroticism factor

Independent variable	Dependent variable	Low			High		
		Beta	T	R^2^	Beta	T	R^2^
Perspicuity	Attitude	0.189*	2.79	0.164	0.027	0.40	0.200
Efficiency		0.198*	2.79		0.346*	5.56	
Dependability		0.110	1.48		− 0.017	− 0.30	
Stimulation		0.270*	3.86		0.220*	2.70	
Novelty		0.065	0.86		0.174*	2.84	
Attitude	Intention to use	0.540*	8.40	0.291	0.476*	6.74	0.227

Significant at *p < 0.05

**Table 9:** Causal relations openness factor

Independent variable	Dependent variable	Low			High		
		Beta	T	R^2^	Beta	T	R^2^
Perspicuity	Attitude	0.119	1.83	0.184	0.181*	2.43	0.171
Efficiency		0.251*	3.95		0.278*	3.78	
Dependability		0.084	1.29		0.018	0.29	
Stimulation		0.262*	3.41		0.245*	3.55	
Novelty		0.177*	2.62		− 0.002	− 0.03	
Attitude	Intention to use	0.453*	6.55	0.205	0.553*	9.07	0.306

Significant at *p < 0.05

Starting with participants with personality traits characterised by Extraversion (Rating of the items: I see myself as someone who is reserved (R) and … is outgoing, sociable). The high-extraversion subsample, characterised by a greater sensitivity towards obtaining rewards and seeking higher social status, explained a higher proportion of Attitude towards using a social robot, reaching an *R*^2^ = 0.19, than the low-extraversion group, with an *R*^2^ = 0.15. In the case of the relationship between attitude and intention to use, the explanatory power is somewhat higher for the low-extraversion (*R*^2^ = 0.28) than for the high-extraversion (*R*^2^ = 0.22). The low model has a 6% better goodness of fit for intention to use than the high model (*R*^2^_*Low*_*–R*^2^_*High*_ = 0.06). This difference could be seen as an estimator of the size of the low extroversion group’s moderation effect relative to the high extroversion group [100]. The main direct drivers for the high-extraversion are Stimulation (*β* = 0.32, *p* < 0.05) and Efficiency (*β* = 0.26, *p* < 0.05) and, with slightly less weight, Perspicuity (*β* = 0.13, *p* < 0.05). In contrast, for the low-extraversion, the weight of the main drivers is the opposite: Efficiency (*β* = 0.27, *p* < 0.05) and Stimulation (*β* = 0.18, *p* < 0.05) and, with slightly less weight, Dependability (*β* = 0.16, *p* < 0.05). That is, although the main driver in the high-extraversion is emotional, the weight of the two functional factors is somewhat higher than the emotional stimulus. Although the low-extraversion reproduce a similar pattern, they do so with the Dependability driver, which has a more functional than cognitive component.

With respect to the Agreeableness profile (Rating of items: I see myself as someone who is generally trusting and … tends to find fault with others (R), … is considerate and kind to almost everyone), again, in this case the more marked personality captures the essence of the model somewhat better. Thus, the high-agreeableness group explains a higher proportion of Attitude, reaching an *R*^2^ = 0.17, than the low-agreeableness group, with an *R*^2^ = 0.15. Furthermore, in the case of the relationship between attitude and intention to use, the explanatory power is somewhat higher for the high-agreeableness (*R*^2^ = 0.25) than for the low-agreeableness (*R*^2^ = 0.23), (*R*^2^_*High*_*–R*^2^_*Low*_ = 0.02). The main direct drivers for high-agreeableness, a trait linked to a predisposition towards altruism and empathy as well as the ability to understand the emotions of others, are Stimulation (*β* = 0.27, *p* < 0.05) and Efficiency (*β* = 0.26, *p* < 0.05) and, with slightly less weight, Perspicuity (β = 0.15, *p* < 0.05). In contrast, for the low-agreeableness, the weight of the main drivers is the opposite: Efficiency (β = 0.26, *p* < 0.05) and Stimulation (β = 0.21, *p* < 0.05) and, with slightly less weight, Novelty (β = 0.18, *p* < 0.05). That is, although the main driver in the high-agreeableness is emotional, the weight of the two functional elements is somewhat higher than the emotional stimulus. However, the low-agreeableness reproduce a completely opposite pattern as they assign a greater weight to the emotional elements.

In terms of the profile of the participants with the most Conscientiousness (Rating of items: I see myself as someone who tends to be lazy (R) and … does a thorough job), the high-conscientiousness subsample explains a somewhat higher proportion of attitude variability, with an *R*^2^ = 0.22, than the low-conscientiousness subsample, with an *R*^2^ = 0.15. Furthermore, in the case of the relationship between attitude and intention to use, the explanatory power is somewhat higher in high-conscientiousness (*R*^*2*^ = 0.27) than in low-conscientiousness (*R*^2^ = 0.23), (*R*^2^_*High*_*–R*^2^_*Low*_ = 0.04). The main direct drivers of the high-conscientiousness subsample, who are oriented towards achieving medium-term goals and delaying short-term gratifications, were Efficiency (*β* = 0.33, *p* < 0.05) and Stimulation (*β* = 0.29, *p* < 0.05) and, with slightly less weight, Perspicuity (*β* = 0.15, *p* < 0.05). In contrast, for the low-conscientiousness subsample, the weight of the main drivers is Novelty (*β* = 0.23, *p* < 0.05), Stimulation (*β* = 0.21, *p* < 0.05), Efficiency (*β* = 0.17, *p* < 0.05) and, with less weight, Dependability (*β* = 0.13, *p* < 0.05). In other words, the main drivers for high-conscientiousness are functional and cognitive factors, while for low-conscientiousness they are emotional factors.

Regarding the Neuroticism profile (Rating items: I see myself as someone who is relaxed, handles stress well (R) and … gets nervous easily), the high-neuroticism group explains a higher proportion of Attitude towards using a social robot, *R*^2^ = 0.20, than the low-neuroticism group, with an *R*^2^ = 0.16. Furthermore, in the case of the relationship between attitude and intention to use, the explanatory power is somewhat higher for low-neuroticism (*R*^*2*^ = 0.29) than for high-neuroticism (*R*^2^ = 0.22); (*R*^2^_*Low*_*–R*^2^_*High*_ = 0.07). The main direct drivers for high-neuroticism, which is characterised by a predisposition to exaggerate negative feelings and a higher sensitivity to threats, are Efficiency (β = 0.34, *p* < 0.05) and Stimulation (β = 0.22, *p* < 0.05) and, with slightly less weight, Novelty (β = 0.17, *p* < 0.05). For low-neuroticism the weight of the main drivers is Stimulation (β = 0.27, *p* < 0.05), Efficiency (β = 0.19, *p* < 0.05) and, with slightly less weight, Perspicuity (β = 0.18, *p* < 0.05). In other words, the main drivers for high neuroticism are emotional factors, where novelty seems important, while for low neuroticism functional and cognitive factors are important.

And finally, there is the profile Openness to new experiences (Rating of the items: I see myself as someone who has few artistic interests (R) and … has an active imagination). The high-openness group explains a lower proportion of Attitude, with an *R*^2^ = 0.17, than the low-openness group, with an *R*^2^ = 0.18. Furthermore, in the case of the relationship between attitude and intention to use, the explanatory power is somewhat lower for low-openness (*R*^2^ = 0.20) than for high-openness (*R*^2^ = 0.30); (*R*^2^_*High*_*–R*^2^_*Low*_ = 0.10). The main direct drivers for high-openness, characterised by a greater openness to new experiences and a bright imagination, are Efficiency (β = 0.27, *p* < 0.05) and Stimulation (β = 0.24, *p* < 0.05) and, with slightly less weight, Perspicuity (β = 0.18, *p* < 0.05). For low-openness the weight of the main drivers is Stimulation (β = 0.26, *p* < 0.05), Efficiency (β = 0.25, *p* < 0.05) and, with slightly less weight, Novelty (β = 0.17, *p* < 0.05). Undoubtedly, one of the most surprising results, as the most imaginative, the high-openness group, grant greater value to the functional-cognitive drivers, while the low-openness value the cognitive factors.

All of this evidence indicates that participants' stated personality affected their appraisal of the service experience delivered by the social robot and its main antecedents. Thus, in four of the five profiles, participants with the most pronounced personality traits achieved a somewhat higher fit than participants with the least pronounced personality traits in explaining attitude.

## Discussion and Conclusions

The provision of services mediated by social robots is becoming increasingly popular among customers in a variety of organisations and businesses, especially after the boost that the emergence of Covid-19 has represented in the implementation of social robots in service organisations [3]. Although a large number of consumers are used to interacting with digital conversational applications (Apps or chatbots), conversations with social bots are not as frequent. In fact, less than half of the robots analysed by Aymerich-Franch and Ferrer [3] had the ability to converse with customers. In this paper we have addressed this issue, as it has focused on analysing the experience of receiving a front-office service from a humanoid social robot, which, equipped with a pre-programmed social intelligence protocol, appears to follow a conversation by offering functional assistance and empathic messages to generate emotions. Functional support is provided by the robot's prompts to participants to complete the game sequence correctly, and emotional support is provided by messages of encouragement and support during the game. We then discuss the theoretical and managerial implications of our findings.

An experiment has analysed a service prototype, measuring the experiences of a sample of users, as well as exploring how consumers' personalities moderate this valuation. Four contributions to the literature are hereby made.

First, the attention offered by a social robot generates positive ratings on both functional and socio-emotional elements. Similar results have been reported in human-delivered services, where emotional support is essential to achieve positive consumer ratings of service quality [39] and in robot-delivered services [101]. That is, the use of social intelligence protocols to create the impression that the robot has conversational skills, capable of issuing advice and expressions of empathy, has contributed to the shaping of the experience.

Second, the holistic and multidimensional nature of the experience of interacting with a social robot has been demonstrated. Although the concept of service experience has been under development for two decades [43], there are no agreed-upon instruments for its measurement, nor is there much empirical research available in the context of service delivery [90], and much less so in service robotics [31, 101].

Third, the User Experience Questionnaire scale, proposed to assess the experience with technology-based interactive products, has been validated [48]. This scale contains both cognitive-functional and emotional factors, which makes it valid for measuring the experience of front-office services. In fact, the results indicate that Efficiency is the main predictor to explain the formation of attitude, which contrasts with the study by Gerlowska et al. [101], who with a sample of elderly people with memory problems reported it as the least relevant factor. In contrast, the Perspicuity factor, the second significant cognitive-functional factor, is among the ones that achieved the highest weight in the study by Gerlowska et al. [101]. The Dependability factor, which was not significant in our study, was one of the most relevant factors in the aforesaid study by Gerlowska et al. [101]. Finally, the relevance of emotional factors, both Stimulation and, to a lesser extent, Novelty, was similar to the study by Gerlowska et al. [101]. In short, although with a slightly lower weight, socio-emotional drivers played a similar role to cognitive-functional drivers in shaping the experience. The results, although not directly comparable to those of Gerlowska et al. [101], seem to indicate that the type of robot, either the prototype version of the Robotic Assistant for Patients with MCI at Home (RAMCIP) or TIAGo, as well as the type of service, a prototype of a customer service assistant, affect the configuration of the experience.

Fourth, it has also been possible to explore how different personality traits, characterised by the two opposite poles (High vs. Low profile), generate different evaluations of the experience, establishing trends that guide possible lines of research. The results show that the model explaining service experience fits best for personality profiles located at the High pole (four out of five) to explain attitude changes, but the transformation from attitude to intention to use is best fit by those in the Low profile (three out of five). For example, the literature reports that extroverts are more willing to interact with social robots [16–18] and, moreover, that the HRI experience generates a significant change in their attitude [70]. But in our study, we have further specified that stimulation was the main driver to explain this change in attitude among more extroverted subjects, but, in line with Cruz-Maya and Tapus [68], we have also highlighted that for the less extroverted, efficiency was the main driver of attitude change. This study also shows that for the less extroverted, this change in attitude is somewhat more likely to be transformed into intention to use. Regarding the Agreeableness and Conscientiousness profiles, the least used in HRI [16], the data from our study also indicates that they are the lowest probability of being considered as moderators. However, for Concientiousness profile, evidence has been collected that the most conscientious subjects follow the robot’s instructions, thus contributing to better task performance [69], and show a greater willingness to use them [72]. This study partially corroborates these points, since one of the main drivers to explain the attitude of the most conscientious subjects is Efficiency, although the greatest shift in attitude towards intention is observed in the least conscientious participants. In turn, Neuroticism is one of the most studied traits in HRI [16], as it usually plays an antagonistic role [18], and, in this study, is most likely to act as a moderator. In fact, for those low in neuroticism, perspicuity is a particularly strong driver to explain attitude in comparison to more neurotic subjects. However, in line with de Cruz-Maya and Tapus [70], efficiency is the main driver to explain the attitude of the most neurotic subjects towards a service provided by a robot. Finally, Openness has been used discretely as a moderator in HRI [16] and, in our study, it is the most likely profile to be used as a moderator. Previous studies have shown that more open user profiles tend to express greater acceptance of social bots, and a stronger belief that their use would improve their performance [72]. This argument is supported by our own results, where both Efficiency and the weight of attitude on the intention to use are somewhat higher in the more open than in the less open group.

In short, the results suggest that consumers' personality profiles, at their extreme poles, moderate their experience with social robots when they provide front-office services. However, given the exploratory nature of this study, these initial results are a basis for the development of further studies to corroborate them.

Regarding the managerial implications, the central conclusion of this study is that the design, use and implementation of social robots with the ability to establish a short conversation, that is, to provide feedback with advice and empathic messages to customers while they are completing a service-related task (completing the documentation during a hotel check-in or processing a transfer through an ATM) contributes to improving the evaluation of the experience and comparing it to that of humans. In line with what was pointed out by Wirtz et al. [10], social robots can be used in a variety of services where simple socio-emotional tasks are required, regardless of the cognitive-analytical complexity of the task.

## Limitations

This study has some limitations. First, this research provides a starting point for understanding the process of evaluating the experience of services provided by social robots. The study has been limited to a simulated service, so other services and different scenarios could provide complementary information that corroborates the results achieved in this research.

Second, a model consisting of five drivers has been validated and only one driver did not obtain a significant result in the general model. However, all the drivers have had a relative importance in the subsamples of personalities, so it is worth testing whether this model can be extended by including some other drivers that would help to increase the explanatory power of the model. Future research could also examine whether the experience of receiving a service delivered by a social robot has other effects in the medium and long term, since the results of this study are but a first experience.

Addressing these issues can help improve our understanding of the HRI experience in a more comprehensive and compelling way. Does the personality of consumers influence their assessment of the experience of interaction with social robots? The results seem to suggest that some personality profiles are more likely to be considered moderators than others. Overall, most of the most commonly used profiles in the HRI literature are confirmed in this study as the most likely to be considered moderators. However, although much effort has been made in this study to obtain a large enough sample, it would still be useful to carry out further studies with even larger samples to corroborate these results. It would also be useful to use the Bonferroni correction to calculate the significant differences between the considered groups, depending on the number of tests carried out. In addition, the personality profile has been analysed without taking into account other complex factors such as the user’s mood, memory and maturity, etc. Finally, it would be interesting to conduct experiments with more homogeneous user profiles in order to obtain more conclusive results. Therefore, we invite further research to help understand how different consumer personalities affect HRI experiences.

## Data Availability

The datasets generated during and/or analysed during the current study are available from the corresponding author on reasonable request.
